# Regulation of Sucrose non-Fermenting Related Kinase 1 genes in *Arabidopsis thaliana*

**DOI:** 10.3389/fpls.2014.00324

**Published:** 2014-07-10

**Authors:** Sarah P. Williams, Padma Rangarajan, Janet L. Donahue, Jenna E. Hess, Glenda E. Gillaspy

**Affiliations:** ^1^Department of Biochemistry, Virginia TechBlacksburg, VA, USA; ^2^Alliance BiosciencesRichmond, VA, USA

**Keywords:** SnRK1, trehalose, sugar-sensing, energy, gene expression

## Abstract

The Sucrose non-Fermenting Related Kinase 1 (SnRK1) proteins have been linked to regulation of energy and stress signaling in eukaryotes. In plants, there is a small *SnRK1* gene family. While the *SnRK1.1* gene has been well studied, the role other SnRK1 isoforms play in energy or stress signaling is less well understood. We used promoter:GUS analysis and found *SnRK1.1* is broadly expressed, while *SnRK1.2* is spatially restricted. *SnRK1.2* is expressed most abundantly in hydathodes, at the base of leaf primordia, and in vascular tissues within both shoots and roots. We examined the impact that sugars have on *SnRK1* gene expression and found that trehalose induces *SnRK1.2* expression. Given that the SnRK1.1 and SnRK1.2 proteins are very similar at the amino acid level, we sought to address whether SnRK1.2 is capable of re-programming growth and development as has been seen previously with SnRK1.1 overexpression. While gain-of-function transgenic plants overexpressing two different isoforms of SnRK1.1 flower late as seen previously in other SnRK1.1 overexpressors, SnRK1.2 overexpressors flower early. In addition, SnRK1.2 overexpressors have increased leaf size and rosette diameter during early development, which is the opposite of SnRK1.1 overexpressors. We also investigated whether SnRK1.2 was localized to similar subcellular compartments as SnRK1.1, and found that both accumulate in the nucleus and cytoplasm in transient expression assays. In addition, we found SnRK1.1 accumulates in small puncta that appear after a mechanical wounding stress. Together, these data suggest key differences in regulation of the *SnRK1.1* and *SnRK1.2* genes in plants, and highlights differences overexpression of each gene has on the development of Arabidopsis.

## Introduction

The Sucrose non-Fermenting Related Kinase 1 (SnRK1) family of proteins has been linked to regulation of energy metabolism and stress signaling in diverse types of eukaryotes (Halford and Hey, 2009; Coello et al., 2011; Tsai and Gazzarrini, 2014). All plants surveyed contain *SnRK1* genes that are structurally and functionally analogous to their yeast and mammalian counterparts, sucrose non-fermenting 1 (*SNF1*) and AMP-activated protein kinase (*AMPK*), respectively. SNF1, AMPK, and SnRK1 are Ser/Thr protein kinases that are considered to function as fuel gauge sensors that sense cellular carbohydrate status and/or AMP/ATP levels in order to maintain growth in response to available energy (Halford and Paul, 2003; Rolland et al., 2006; Hey et al., 2010; Ghillebert et al., 2011). SnRK1 proteins carry out their function as part of heterotrimeric protein complexes, binding to β and γ subunits (Lumbreras et al., 2001; Gissot et al., 2006; Polge et al., 2008) or to a hybrid βγ subunit in plants (Kleinow et al., 2000; Lopez-Paz et al., 2009; Ramon et al., 2013).

The small Arabidopsis *SnRK1* gene family is composed of three genes, *SnRK1.1*, *SnRK1.2*, and *SnRK1.3* (Baena-Gonzalez et al., 2007). While *SnRK1.3* is considered a non-expressed pseudogene, the roles of *SnRK1.1* and *SnRK1.2* were delineated in seminal work by Baena-Gonzalez using a genetics approach in *Arabidopsis thaliana*. Overexpression of the *SnRK1.1* gene re-programs metabolism such that flowering and senescence of mature plants is delayed (Baena-Gonzalez et al., 2007). The delay in flowering and senescence effectively lengthens the lifespan of the plant, most likely through the combined direct protein phosphorylation of SnRK1 substrates, regulation of transcription in the nucleus (Baena-Gonzalez et al., 2007), and post-transcriptional regulation of target genes (Confraria et al., 2014). Some of the known SnRK1 substrates are key metabolic enzymes such as sucrose phosphate synthase, nitrate reductase, and HMG-CoA reductase (Halford et al., 2003), while others such as FUS3 are transcription factors (Tsai and Gazzarrini, 2012a). FUS3 and SnRK1.1 interact to regulate embryonic-to-vegetative and vegetative-to reproductive phase transitions and lateral organ development (Tsai and Gazzarrini, 2012a). *FUS3*-overexpression delays vegetative growth and flowering by increasing levels of ABA, while repressing GA biosynthesis and ethylene signaling (Gazzarrini et al., 2004). Therefore, SnRK1 may regulate post-embryonic development through regulation of hormone biosynthesis and signaling. Analysis of *SnRK1* genetic mutants in Arabidopsis showed that *SnRK1.1* and *SnRK1.2* genes have partially redundant functions, and when expression of both genes are reduced, seedling growth under low energy conditions is compromised and mature plants undergo growth deprivation, early flowering, and early senescence (Baena-Gonzalez et al., 2007). Comparable phenotypes were also shown in moss (*Physcomitrella patens*), in which a double mutant lacking the two *SnRK1* genes has accelerated development and early senescence (Thelander et al., 2004).

Other gain-of-function studies have shown that SnRK1.1 overexpressors are altered in ABA as well as sugar signaling pathways (Jossier et al., 2009). Recent work has shown that SnRK1.1 and SnRK1.2 interact with two clade A type 2C protein phosphatases, established repressors of the ABA signaling pathway. Inactivation of SnRK1.1 by these phosphatases may allow for the coordinated activation of ABA and energy signaling (Rodrigues et al., 2013). The SnRK1.1 and SnRK1.2 proteins are 81% identical and contain a similar arrangement of functional domains including an N-terminal kinase domain, a Ubiquitin associated (UBA) domain, and a C-terminal kinase associated domain. The regions outside the kinase domain have been shown to facilitate SnRK1 protein interactions with various proteins, indicating that SnRK1 proteins may be part of several different protein complexes (Singh et al., 2009; Ng et al., 2013; Lin et al., 2014; Mohannath et al., 2014).

Interestingly, biochemical data has indicated that SnRK1 activity in plant cells is mostly a function of the SnRK1.1 gene product (Jossier et al., 2009), thus the *SnRK1.2* gene may play a minor or restricted role in regulating most plant metabolism, stress and/or energy sensing. The Arabidopsis *SnRK1.2* gene has been overexpressed in *N. benthamiana*, where overexpression leads to enhanced resistance to geminiviral infection, although at the cost of adverse effects on plant growth (Mohannath et al., 2014). Although the network of genes regulated by SnRK1 has been elucidated (Baena-Gonzalez et al., 2007), our understanding of how the *SnRK1.1* and *SnRK1.2* genes themselves are regulated and function with respect to one another has not been addressed. The *SnRK1* genes have been shown to be expressed throughout development, including within the meristem and leaf primordia (Takano et al., 1998; Pien et al., 2001; Bradford et al., 2003; Fragoso et al., 2009; Bitrian et al., 2011), however very little spatial information on the expression of different *SnRK1* genes within a species exists.

Given that there are multiple cDNAs of both *SnRK1* genes expressed in plants, we sought to address the spatial regulation of *SnRK1.1* and *SnRK1.2* in Arabidopsis, how different sugars impact expression of these genes, and how different SnRK1 protein isoforms alter plant growth and development when overexpressed. Our data show that *SnRK1.2* gene expression is spatially restricted within Arabidopsis, and can be induced by trehalose, but not other sugars. When overexpressed, a SnRK1.2-green fluorescent protein (GFP) gene fusion alters development in a manner opposite in nature to overexpression of *SnRK1.1-GFP*. These data suggest that *SnRK1.2* may have a unique function in plants that warrants further investigation.

## Materials and methods

### Plant growth conditions

*Arabidopsis thaliana* ecotype Columbia and *Landsberg erecta* (Ler) plants were used for all experiments. For seedling growth experiments, seeds were surface-sterilized and plated on 0.5× Murashige and Skoog (MS) salts + 0.8% agar medium plus the indicated sugars. Seed were stratified at 4°C for 3 days and grown under 120–130 μE m^−2^ s^−1^ light under long day (16 h light) conditions with a mixture of fluorescent and incandescent lamps. All plant growth analyses were performed with at least three different biological replicates.

### Construction of promoter-reporter transgenic plants and imaging

The promoter sequences of *SnRK1.1* (At3g01090) and *SnRK1.2* (At3g29160) were analyzed using tools available from the web site Plant Cis-Acting Regulatory Element (P.L.A.C.E.) (Higo et al., 1999). We focused on using the native, intergenic regions of *SnRK1.1* and *SnRK1.2* genes, which is approximately 0.8 and 4.3 kB of the *SnRK1.1* and *SnRK1.2* 5′ upstream sequences (i.e., promoters, respectively). These were amplified from CS60000 genomic DNA by PCR using the primers indicated in Supplemental Table 1, and were cloned via the Gateway system into plasmid pBGWFS7 (Karimi et al., 2002) containing an *eGFP:uidA* gene fusion. GUS constructs were transformed into Arabidopsis using *Agrobacterium* transformation (Bechtold et al., 1993). Homozygous lines were obtained through BASTA resistance screening. GUS staining of 3–10-day old grown on 0.5× MS agar plates + indicated sugars or of plant tissues from soil grown plants has been described (Styer et al., 2004), and staining was observed using an Olympus SZX16 and Zeiss Axiophot microscope. At least three biological replicates of different developmental stage and sugar-treated seedlings were performed.

### Quantitative PCR

Conditions have been previously described (Donahue et al., 2010). Briefly, RNA was purified using a Qiagen RNeasy kit with DNase treatment, from 6 week-old soil-grown plants (roots, leaves, cauline leaves, flowers, siliques). Silique RNAs were initially extracted with phenol/chloroform and precipitated with LiCl before RNeasy purification. RNA was also extracted from seedlings grown on 0.5× MS agar plates for 5 days or 10 days. cDNA was synthesized from RNA using a Bio-Rad iScript cDNA synthesis kit. Reactions containing cDNA, Sybr Green MasterMix (Applied Biosystems) and primers, were performed in triplicate (61°C annealing temperature) and monitored with Applied Biosystems 7300 Real-Time PCR instrumentation outfitted with SDS software version 1.4. Primers were designed to detect the longest SnRK1.1 cDNA (Accession no. AY093170), all known cDNAs encoding SnRK1.1, SnRK1.2 cDNA, and PEX4 cDNA (At5g25760) (Supplemental Table 1). For all experiments, at least two biological replicates were performed.

### Flowering time and leaf size assays

WT and mutant plants were grown as described previously under long-day (16 h days) conditions. Careful attention was given to growing plants side-by-side or in the same pot for comparison. Plants were examined at the point of inflorescence emergence and leaves were counted. Three biological replicates were analyzed. For leaf measurements the length and width of the largest leaf on each 27 d soil-grown plant was measured (*N* = 100–120 plants per genotype).

### Protein blot analyses

Conditions have been previously reported (Burnette et al., 2003). Briefly, tissues were ground in liquid nitrogen, homogenized, and resuspended with a pestle in SDS-bromophenol blue loading dye, boiled, and the supernatant was loaded onto a polyacrylamide gel for separation. Equal amounts of protein were loaded onto gels. SDS-PAGE was followed by western blotting with a 1:20,000 dilution of rabbit anti-GFP antibody (Invitrogen Molecular Probes, Eugene, OR) or an anti-SnRK1.1 antibody. This antibody was produced by CoCalico, Inc. by injecting rabbits with a SnRK1.1-V5 recombinant protein (Burnette et al., 2003) purified by ion-metal affinity (IMAC) and size exclusion chromatographies (Supplemental Figure 2). Sera from rabbits was purified by affinity-blot purification (as described in Harlow and Lane, 1988) and then tested for specificity with protein blots containing recombinant SnRK1.1, or extracts from SnRK1.1T-HA and previously characterized SnRK1.1 RNAi knock-down lines (Baena-Gonzalez et al., 2007) (Supplemental Figure 2). This anti-SnRK1.1 antibody does not cross-react with SnRK1.2-GFP. A secondary goat, anti-rabbit horseradish peroxidase antibody (Bio-Rad Laboratories, Hercules, CA) was used at a 1:2500 dilution. Immunoreactive bands were detected using an ECL Plus Western Blotting Detection System (Amersham, Buckinghamshire, UK). Ponceau S staining of blots was performed to ensure that equal amounts of protein within extracts were analyzed.

### SnRK1 activity assays

The immunoprecipitation reactions were carried out as described (Ercetin et al., 2008) with the following modifications: leaves from mature 60 d plants were ground in liquid nitrogen and resuspended in extraction buffer (50 mM Tris-Cl, pH7.5, 150 mM NaCl, 1 mM EDTA, 10 mM MgCl_2_, 0.1% Triton X-100, 10% glycerol) containing protease inhibitor (Sigma 9599), 10.5 μM MG132 (proteasome inhibitor), and 10 mM DTT. After centrifugation at 13.2 rpm for 15 min the supernatant was incubated with either anti-GFP agarose or anti-HA antibody bound to protein A sepharose beads. Extracts and beads were rocked for 3 h at 4°C, then washed 4× with Buffer A and 2× with 50 mM HEPES, pH 7, 1 mM DTT. After washing, these beads were incubated in SnRK1 activity assays as described in Ananieva et al. (2008). Briefly, beads were incubated in kinase buffer (50 mM HEPES and 1 mM DTT, pH 7.0), with SPS substrate peptide and γ−^32^P-ATP, unlabeled ATP, and MgCl_2_. Samples were incubated for 30 min at 30°C, and spotted onto P81 paper and washed in 125 mM phosphoric acid as described. A reaction control with no SPS peptide to correct for autophosphorylation, and a no protein extract control were included. Activity is expressed as pmoles of phosphate incorporated into peptide per reaction. The assay was performed using two biologically independent extracts and three replicates of each extract. An initial time course with immunoprecipitated proteins indicated that product accumulation was linear over 45 min in these assay conditions (Supplemental Figure 2). Two biological replicates were analyzed.

### GFP localization and imaging

The coding regions of *SnRK1.1*, *SnRK1.1T*, and *SnRK1.2* were amplified from plasmids or 7 d seedlings using the primers indicated in Supplemental Table 1, and were recombined into vector pK7FWG2 (Karimi et al., 2002) containing the 35S Cauliflower Mosaic virus promoter and a C-terminal *GFP* gene. Transformation of Arabidopsis was as described (Bechtold et al., 1993). Mature leaves of homozygous Arabidopsis lines were used for GFP fluorescence detection using a Zeiss LSM 510 laser-scanning microscope (Carl Zeiss) with an inverted Axio Observer Z1 base. GFP excitation was done using a 488-nm argon laser and fluorescence detected using 505- to 550-nm band-pass emission filter. Slides were examined with ×40 C-Apochromat water immersion lens. *Nicotiana benthamiana* plants were agro-infiltrated as previously described (Kapila et al., 1997). A set of mCherry tagged organelle markers were used for co-localization experiments (Nelson et al., 2007). Leaf sections were imaged 48 h post infiltration using the confocal microscope described above. mCherry was imaged using excitation with a 543-nm HeNe laser and 560-nm band-pass emission filter. At least three biological replicates were analyzed for each.

## Results

### Developmental regulation of SnRK1.1 and SnRK1.2 expression

**Figure 1:**
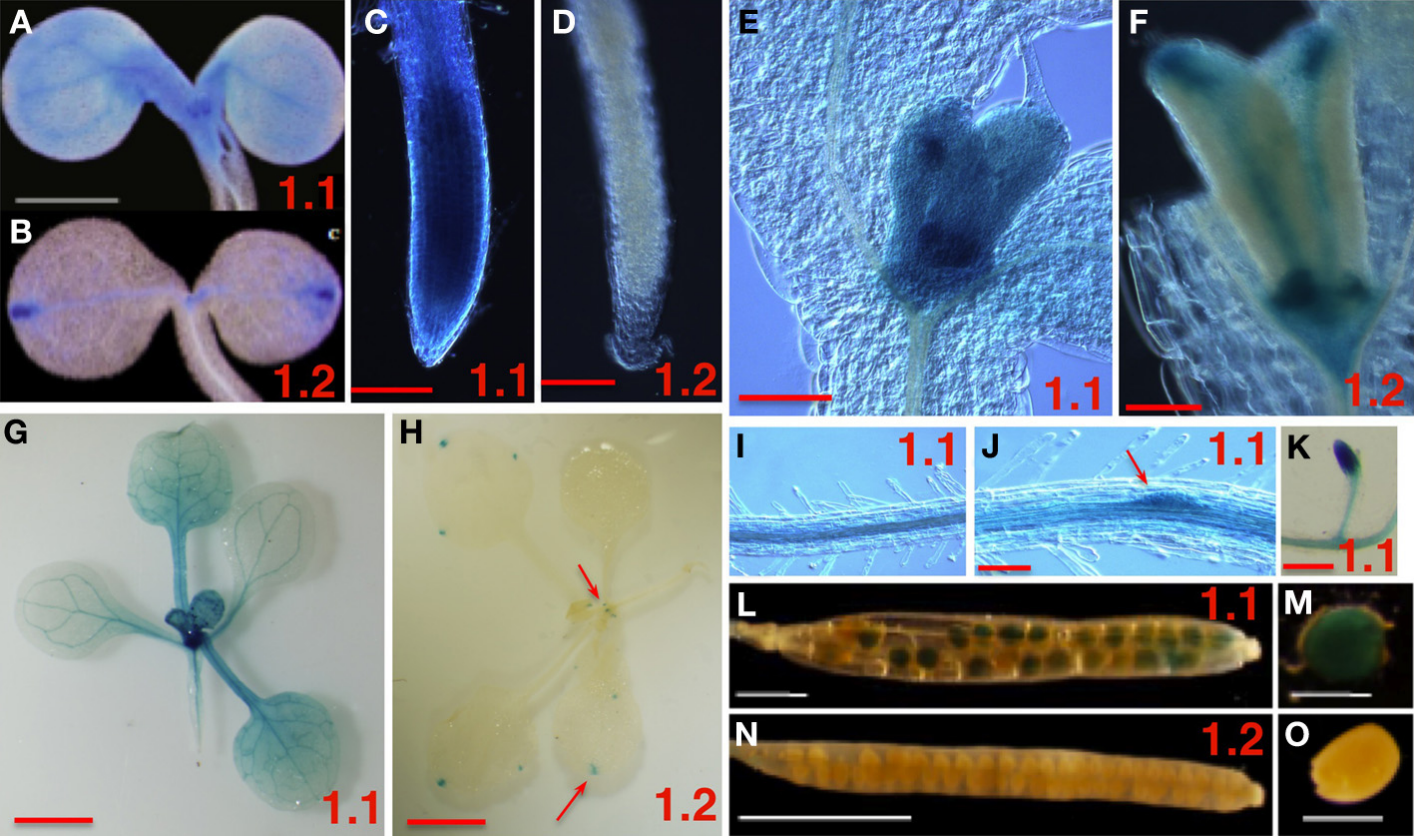
**Spatial Expression Patterns of *SnRK1.1* and *SnRK1.2* Genes**. The promoters from *SnRK1.1* (1.1 shown in **A,C,E,G,I–M**) or *SnRK1.2* (1.2 shown in **B,D,F,H,N,O**) were used to drive GUS expression in transgenic plants. **(A–F,I,J)** are from plants grown on MS agar with no added sugar, and **(G,H,L–O)** are from soil-grown plants. **(A,B)** 5 d cotyledons, bar = 1 mm. **(C,D)** 5 d roots, bar = 100 μM. **(E,F)** 10 d leaf primordia, bar = 100 μM. **(G,H,K)** 15 d plants and roots, bar = 2 mm. **(I,J)** 10 d roots, bar = 100 μM; **(L,M)** siliques, **(N,O)** developing seed, bar = 500 μM. The red arrows indicate small areas which are positive for staining.

**Figure 2:**
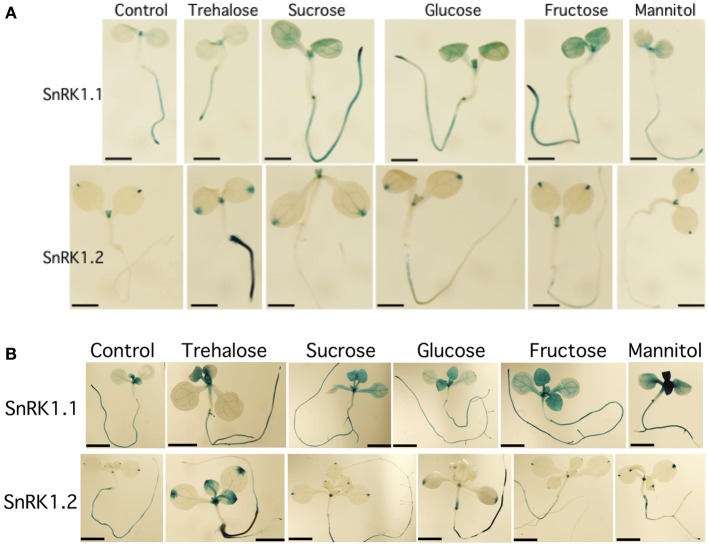
**Effects of sugars on the expression of *SnRK1.1* and *SnRK1.2*. (A)** 5 d seedlings grown with 1% trehalose, 3% sucrose, 1.5% glucose, 1.5% fructose or 3% mannitol. Bar = 1 mm. **(B)** 10 d seedlings grown with the same sugars as in **(A)**. Bar = 2 mM.

To explore the role SnRK1 may play in plant growth and development, we examined the spatial and temporal regulation of expression of the *SnRK1.1* and *SnRK1.2* genes. Promoter:gene reporter transgenic plants were constructed using 0.8 and 4.3 kB of the *SnRK1.1* and *SnRK1.2* promoters, respectively. Multiple lines of *SnRK1.1p:GUS* and *SnRK1.2p:GUS* seedlings were identified, and homozygous lines were isolated and studied to verify reproducibility of GUS staining patterns. When *SnRK1.1p:GUS* and *SnRK1.2p:GUS* seedlings are grown on MS agar without an added carbon source, a striking difference in GUS expression is noted (Figures 1A–D). The *SnRK1.1* promoter drives expression throughout the seedling, with highest expression in leaf primordia and vascular tissue (Figure 1A), and the seedling root tip (Figure 1C). In contrast, expression of the *SnRK1.2* promoter is restricted to only a few cells at this stage including hydathodes, leaf primordial, and in portions of the cotyledon vascular tissue (Figures 1B,D). At 10 days both promoters drive strong expression in developing leaves (Figures 1E,F), with the *SnRK1.2* promoter restricted to the base of the leaf, the vascular tissue, and the hydathodes (Figure 1F). Soil-grown plants were analyzed and indicate that *SnRK1.1* continues to be broadly expressed in the shoot (Figure 1G), whereas, activity of the *SnRK1.2* promoter is restricted to the base of newly developing leaves and hydathodes (Figure 1H). In roots, expression of *SnRK1.1* is abundant in vascular tissue and developing lateral root primordia with no added carbon source (Figures 1J,K, 2A,B), and in soil (Figure 1K). *SnRK1.2* is not abundantly expressed in roots until 10 d, at which time its expression is similar to that of *SnRK1.1* (Figures 2A,B). *SnRK1.1* is expressed in developing embryos within siliques, but *SnRK1.2* is not (Figures 1L–O). We conclude that *SnRK1.1* is more abundant and broadly expressed in plant tissues, whereas *SnRK1.2* expression is more spatially restricted.

### Regulation of SnRK1.1 and SnRK1.2 expression by sugars

**Figure 3:**
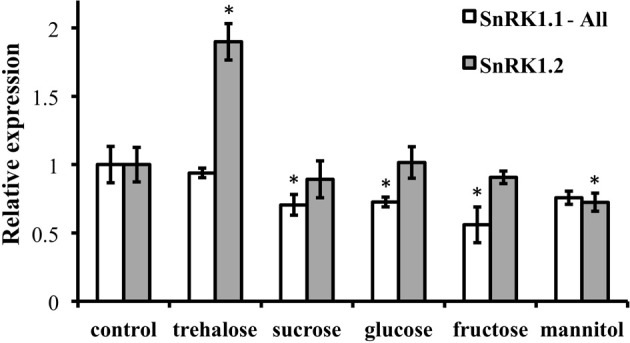
**RNA levels of *SnRK1.1* and *SnRK1.2* in sugar-treated seedlings**. Wildtype seedlings were grown for 9 days and then treated with no sugar (control), 1% trehalose, 1.5% sucrose, 1.5% fructose, or 3% mannitol. *SnRK1.1* and *SnRK1.2* expression was measured with real-time PCR and was normalized to *SnRK1.1* control and *SnRK1.2* control levels, respectively, which are set to 1. SnRK1.1-All primers amplify all SnRK1.1 cDNAs, including those that encode SnRK1.1 and SnRK1.1T proteins. Means of triplicate reactions ± SD are presented. ^*^p-value ≤ 0.05 when compared to the control.

Because of the role of SnRK1 in carbon sensing and allocation in plants (Baena-Gonzalez et al., 2007; Jossier et al., 2009; Cho et al., 2012), we investigated whether different carbon sources would alter the patterning of *SnRK1* expression as measured in *SnRK1.1p:GUS* and *SnRK1.2p:GUS* plants. In addition, we used quantitative RT-PCR (Q-PCR) to determine whether the abundance of *SnRK1.1* and *SnRK1.2* transcripts in wildtype plants were increased by sugar treatment. Seedlings grown for 5 and 10 d in the presence of trehalose, sucrose, glucose, fructose, or mannitol, did have small alterations in expression of *SnRK1.1* in the shoot, but these changes may be indicative of changes in the developmental state (see GUS staining in cotyledons and new leaves in Figures 2A,B top panels). When a shorter-term treatment of sugar (24 h) is given to 9 d seedlings and *SnRK1.1* expression is measured by Q-PCR, we find that *SnRK1.1* expression is slightly, but significantly, decreased by treatment with fructose, glucose, sucrose, but not trehalose or mannitol (Figure 3). In contrast, trehalose increases *SnRK1.2* expression, as seen by changes in GUS staining of *SnRK1.2p:GUS* seedlings grown for 5 or 10 d in trehalose (Figures 2A,B). Specifically, trehalose induced expression of *SnRK1.2* in 5 d seedling roots that extended to the top of the root tip (Figure 2A, bottom panel). Trehalose also elevated *SnRK1.2* expression in 10 d seedling roots, hydathodes, vascular tissue, and the base of the leaf (Figure 2B, bottom panel). This increase in *SnRK1.2* expression is also noted when a shorter-term treatment of sugar (24 h) is given to 9 d seedlings (Figure 3). In this short-term sugar treatment, mannitol also decreased *SnRK1.2* expression to small degree (Figure 3). We conclude that trehalose increases *SnRK1.2* expression, while mannitol decreases *SnRK1.2* expression. Regulation of *SnRK1.1* differs as other sugars such as sucrose, glucose, and fructose result in a small decline in *SnRK1.1* expression in seedlings.

### SnRK1.1 and SnRK1.2 cDNAs and SnRK1 protein isoforms

**Figure 4:**
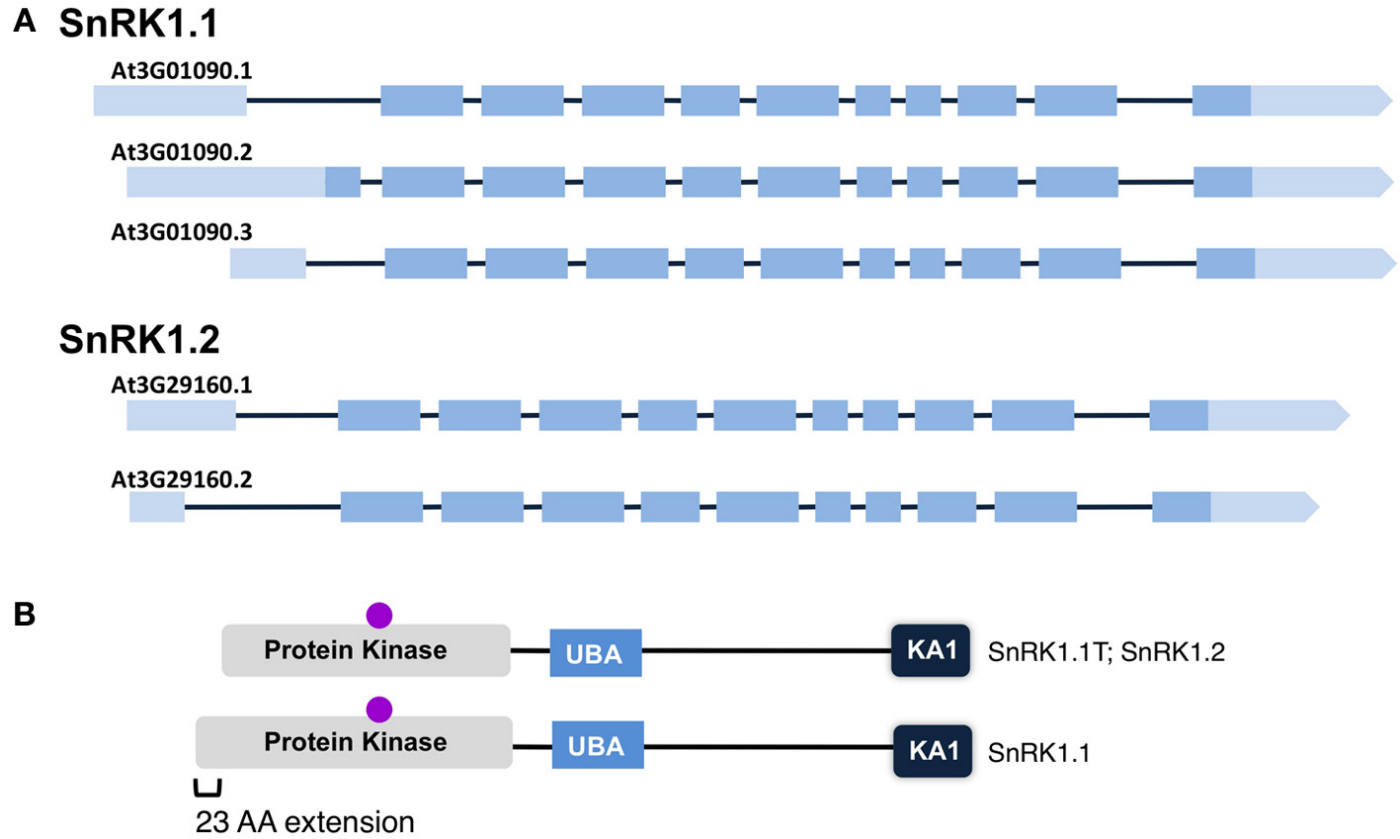
**Maps of SnRK1 Isoforms and Domain Structures. (A)** The reported intron-exon maps of *SnRK1* genes. Dark boxes denote exons while light boxes denote 5′ and 3′ UTRs. **(B)** SnRK1 kinases contain 3 domains: Protein Kinase, Ubiquitin Associated (UBA), and Kinase Associated 1 (KA1). The location of the active site residues are indicated by a purple circle.

After noticing significant differences in a group of so-called full-length *SnRK1.1* cDNAs, we compared the structures of different *SnRK1.1* and *SnRK1.2* cDNAs (Figure 4A). For this analysis we focused solely on verified full-length cDNAs. Both *SnRK1* genes are predicted to undergo differential splicing of a non-coding exon located at the 5′end, and in the case of *SnRK1.1*, alterations in splicing can result in two SnRK1.1 protein isoforms differing in a 23 amino acid extension at the N-terminus (Figures 4A,B). We have named the shorter truncated protein SnRK1.1T, while we call the longer protein SnRK1.1 (Figure 4B). Comparison of SnRK1.1, SnRK1.1T, and SnRK1.2 predicted proteins shows that the 23 amino acid extension is unique to SnRK1.1, while all three proteins contain highly similar sequences and protein domains (Figure 4B, Supplemental Figure 1). It is important to note that most investigations on *SnRK1.1* focus on the SnRK1.1T isoform, including the complementation of the yeast *snf1* mutant (Alderson et al., 1991), and re-programming of Arabidopsis growth and ABA responses by overexpression (Baena-Gonzalez et al., 2007; Jossier et al., 2009).

**Figure 5:**
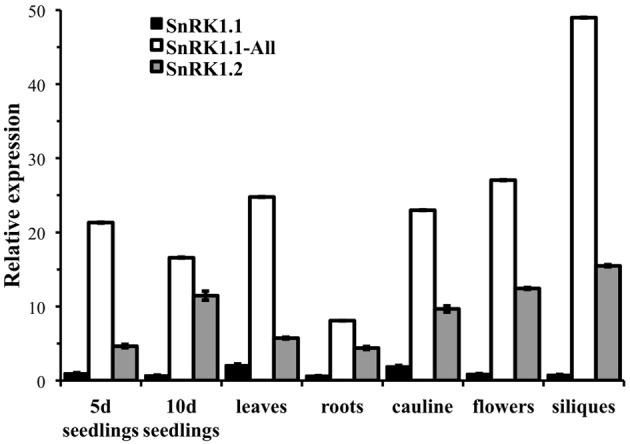
**Relative expression of *SnRK1* genes as determined by real-time PCR**. Primers specific for amplifying the longer cDNA clone that encodes the SnRK1.1 protein (SnRK1.1), all SnRK1.1 cDNAs (SnRK1.1—All), and SnRK1.2 were used to measure expression in 5 d and 10 d seedlings (seedlings), and tissues from 6 week-old soil-grown plants: rosette leaves, roots, cauline leaves, flowers, and green siliques. *SnRK1* expression was compared to *PEX4* to generate relative expression levels. Means of triplicate reactions ± SD are presented.

We first used quantitative PCR to confirm the developmental expression patterns noted with promoter-GUS constructs, and to confirm that cDNAs capable of encoding the SnRK1.1 and SnRK1.1T proteins were expressed in Arabidopsis. For this work we designed oligonucleotide primers to amplify the unique 5′end of the longer *SnRK1.1* cDNA that can encode the extra 23 amino acids (SnRK1.1), as well as the set of primers we previously used to amplify ***all*** *SnRK1.1* cDNAs (called SnRK1.1-All), plus a set to amplify *SnRK1.2*. We found very little expression of the longer SnRK1.1 isoform in all tissues examined (Figure 5). In contrast, our SnRK1.1-All primers detected expression at fairly high levels in seedlings, leaves, roots, cauline leaves, flowers, and siliques (Figure 5), indicating that the majority of SnRK1.1 expressed in these tissues has the ability to encode the SnRK1.1T protein isoform. *SnRK1.2* was also expressed in these tissues, but at a lower level as compared to *SnRK1.1* (Figure 5).

### Overexpression of different SnRK1 proteins in planta

**Figure 6:**
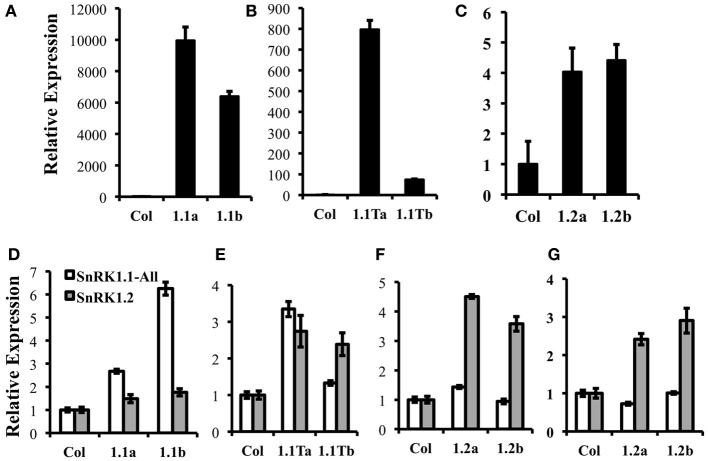
**Expression of Transgenes in SnRK1 transgenic plants. (A–C)** Relative levels of *GFP* expression in 10 d seedlings of wild type (Col), SnRK1.1-GFP, SnRK1.1T-GFP, and SnRK1.2-GFP. **(D–G)** Relative levels of *SnRK1.1* and *SnRK1.2* expression in the indicated lines with RNA extracted from **(D–F)** rosette leaves of 60 d soil-grown plants, and **(G)** 10 d seedlings. Means of triplicate reactions ± SD are presented. Two or more biological replicates were performed for each.

To determine whether the three SnRK1 protein isoforms (SnRK1.1, SnRK1.1T, and SnRK1.2) are each capable of altering plant growth and development, we compared the impact of overexpression of each. We used the 35S CaMV promoter to drive SnRK1-green fluorescent protein (GFP) gene fusions, and identified two independent lines with ectopic expression of *SnRK1.1*, *SnRK1.1T*, and *SnRK1.2*. Transgenic plants were characterized with respect to transgene expression using real time PCR. Figure 6 shows that our SnRK1.1-GFP, SnRK1.1T-GFP, and SnRK1.2-GFP plants accumulate transgenic RNA (Figures 6A–C, respectively). In addition, we examined total *SnRK1.1* and *SnRK1.2* RNAs in these lines to confirm overexpression (Figures 6D–G). This analysis also showed that overexpression of the transgene was greater in some lines as compared to others, and could vary during development. For example, SnRK1.1a contains higher expression of *SnRK1.1* than SnRK1.1b in seedlings, but not leaves (Figures 6A,B,D,E). Interestingly, *SnRK1.2* levels were increased in SnRK1.1T-GFP plants (Figure 6E). (Figures 6F,G) indicate that SnRK1.2-GFP overexpression seedlings have a 2.4- and 2.9-fold increase in *SnRK1.2* RNA (Figure 6F), while leaves from these same plants indicate a 4.5- and 3.6-fold increase in *SnRK1.2* expression (Figure 6G).

**Figure 7:**
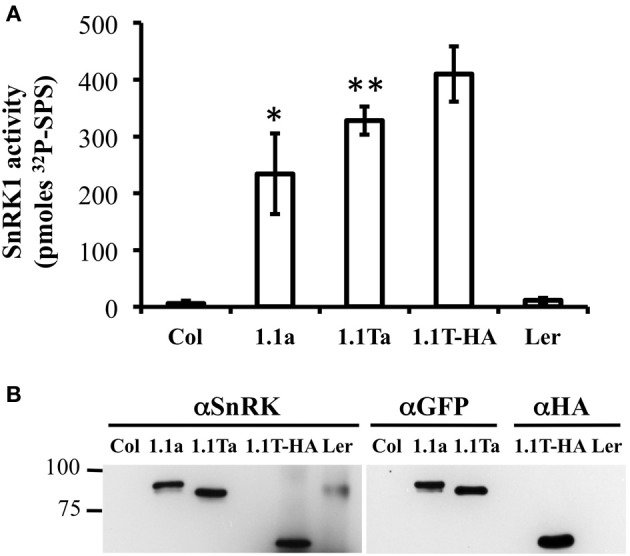
**SnRK1 activity in Transgenic Plants. (A)** SnRK1 proeins were immunoprecipitated as described in the Materials and Methods, and kinase assays were performed. Means of 3 triplicate reactions ± SD are presented. ^**^p-value ≤ 0.01, and ^*^indicates a *p*-value less than 0.05 as compared to the 1.1T-HA sample. **(B)** An equal amount of sample was detected by western analysis with anti-SnRK, anti-GFP, or anti-HA antibody.

We also wanted to verify that our SnRK1 transgenic plants accumulated active SnRK1 protein. Since removal of other contaminating kinases present in plant extracts is key to examining SnRK1 activity, we used immunoprecipitation of tagged SnRK1 proteins from plant extracts, followed by SnRK1 activity assays for this verification. Previously characterized Arabidopsis SnRK1 overexpressors constructed in the *Landsberg erecta* (Ler) ecotype (herein called SnRK1.1T-HA plants) were utilized as a positive control, and wildtype Arabidopsis Col and Ler plants were used as negative controls. Crude protein extracts from leaves were incubated with either anti-GFP agarose or anti-HA antibodies coupled to Protein A sepharose beads, and bound, washed proteins were incubated in SnRK1 activity assays with the SPS substrate peptide and γ-P^32^-ATP as described previously (Ananieva et al., 2008) (Figure 7A). In addition, bound and washed proteins were analyzed by western blotting with anti-SnRK1.1, anti-GFP or anti-HA antibodies to examine the immunoprecipitated proteins (Figure 7B). The anti-SnRK1 antibody is described in detail in the Materials and Methods. As expected, we found that wildtype Col and Ler extracts had minimal (i.e., background) levels of SnRK1 activity (measured by the pmoles of P^32^ added to the SPS substrate peptide) (Figure 7A). In contrast, immunoprecipitated proteins from SnRK1.1-GFP, SnRK1.1T-GFP, and SnRK1.1T-HA plants indicated that all transgenic SnRK1.1 proteins are catalytically active (Figure 7A). However, immunoprecipitated SnRK1.1-GFP has 57%, and SnRK1.1T-GFP has 80%, respectively, of the activity of SnRK1.1T-HA, suggesting that both GFP-tagged proteins have a reduction in their SnRK1 activity as compared to the HA-tagged protein. Western blotting with an anti-SnRK1 antibody verifies that proteins of the correct size were present in the immunoprecipitation, that anti-GFP and anti-SnRK1 antibodies detect similarly sized proteins, and that the SnRK1.1T protein is slightly decreased in size from the larger, SnRK1.1 protein (Figure 7B). It is important to note that we tried several times to immunoprecipitate SnRK1.2-GFP from transgenic extracts, but could not obtain enough protein to do reliable activity assays. We could detect a faint band corresponding to SnRK1.2-GFP in both crude plant extracts and immunoprecipitations, so we conclude that SnRK1.2-GFP protein is being synthesized, however, the levels of this protein are very low, and preclude measuring enzyme activity.

### Overexpression of SnRK1.2 decreases time to flowering

**Figure 8:**
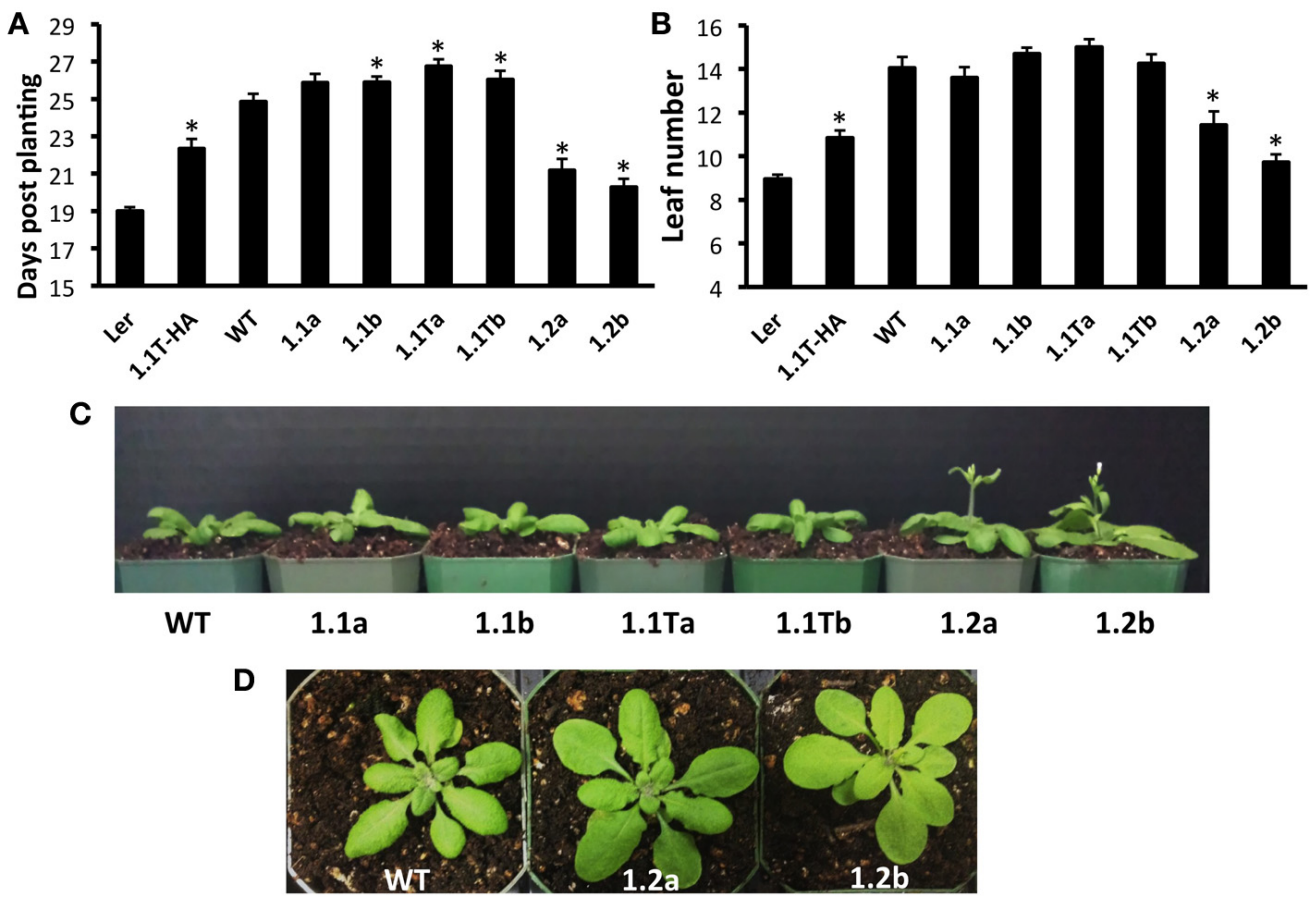
**Flowering Time Alterations in SnRK1 Transgenic Plants. (A)** Days to flowering and **(B)** Number of leaves at flowering of soil-grown Arabidopsis wild type and SnRK1 overexpressing lines. **(C)** 21 d plants **(D)** 23 d plants. *N* = 30 plants for each genotype. Means ± SE are presented. ^*^p-value ≤ 0.05 when compared to the wildtype control. This experiment was replicated three times.

Previously characterized Arabidopsis SnRK1 overexpressers (SnRK1.1T-HA plants constructed in the Ler background) (Baena-Gonzalez et al., 2007), and SnRK1.1-HA plants constructed in the Col background (Tsai and Gazzarrini, 2012a), flower late, due to delayed developmental transitions throughout the life of the plant (Tsai and Gazzarrini, 2012b). This suggests that the 23 amino acid N-terminal extension missing in the SnRK1.1T protein does not impact function. Whether or not overexpression of SnRK1.2 has a similar effect on delaying developmental transitions has not been reported. To address these issues, we compared our SnRK1.1-GFP, SnRK1.1T-GFP, and SnRK1.2-GFP plants to the previously characterized SnRK1.1T-HA plants (see Supplemental Figure 3). We grew each transgenic line and matched wildtypes in different growth rooms and chambers set to standard long-day conditions in three separate experiments, and measured the days to flowering (Figure 8). As shown previously by others, SnRK1.1T-HA plants are significantly delayed in their flowering, by 3.3 d (Figure 8A). Both SnRK1.1-GFP lines also consistently flower late by app. 1 d, and this difference is statistically significant for the SnRK1.1b line (Figure 8A). The SnRK1.1T-GFP lines also flower late by 1.9 and 1.2 d, respectively (Figure 8A). Unexpectedly, both SnRK1.2-GFP lines flower early with a 3.6 and 4.5 d difference in days to flowering, respectively (Figure 8A). These same trends in flowering time were also seen when the number of leaves to flowering were measured, although in some cases the differences were not statistically significant (Figure 8B). We conclude that overexpression of SnRK1.2 has the opposite impact on flowering time as compared to that of SnRK1.1 overexpression. We also conclude that the reduction in SnRK1 activity caused by the GFP tag and the extra 23 aa present in SnRK1.1-GFP do not alter the ability of these proteins to induce late flowering when overexpressed, however, these changes may decrease the degree to which flowering time is delayed.

**Figure 9:**
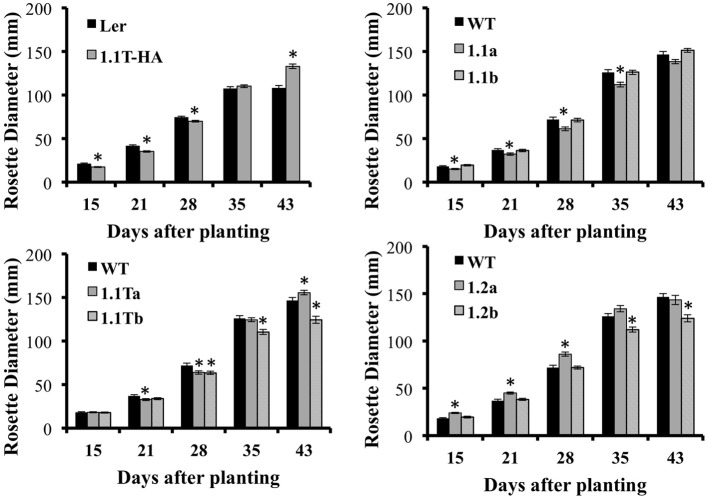
**Rosette Diameter Measurements**. Rosette diameter of soil-grown plants grown under long day conditions for the indicated number of days. Means ± SE are presented. ^*^p-value ≤ 0.05 when compared to the wildtype control.

In addition to flowering early, we also noticed that SnRK1.2-GFP plants tend to have flatter leaves (Figure 8C), that are ~120% longer and 108% wider as compared to wildtype leaves (Supplemental Figure 4). Thus, overall, the appearance of SnRK1.2-GFP plants is altered when compared to wildtype or the other SnRK1.1-GFP lines (Supplemental Figure 4). It is interesting to note that in our hands, the SnRK1.1T-HA plants are developmentally delayed until flowering, and then after flowering vegetative biomass increases, which is a potentially valuable trait (Supplemental Figure 4). To compare the growth and development of all SnRK1 transgenic lines, we examined growth throughout development, using rosette diameter as one indicator of organism size (Figure 9). This analysis shows that SnRK1.1T-HA plants have a smaller rosette size through 28 d, and by 35 d their rosette diameter is larger (Figure 9). SnRK1.1-GFP and SnRK1.1T-GFP lines have a similar trend up to 28 d, with both lines of each type showing smaller rosette diameter (Figure 9). In contrast, both SnRK1.2-GFP lines have a larger rosette diameter until 28 d (Figure 9). After 28 d, data on rosette width starts to vary within each set of lines (Figure 9). Even though we tried to tightly control these experiments and repeated them with 3 biological replicates (*N* = 100–120 plants of each type), we have been unable to obtain consistent results on organism size of SnRK1-GFP plants during late stages of development. This is in stark contrast to the SnRK1.1T-HA plants that show very consistent increases in organismal size post-flowering (Figure 9, Supplemental Figure 4).

### Subcellular localization of SnRK1.1, SnRK1.1t and SnRK1.2

**Figure 10:**
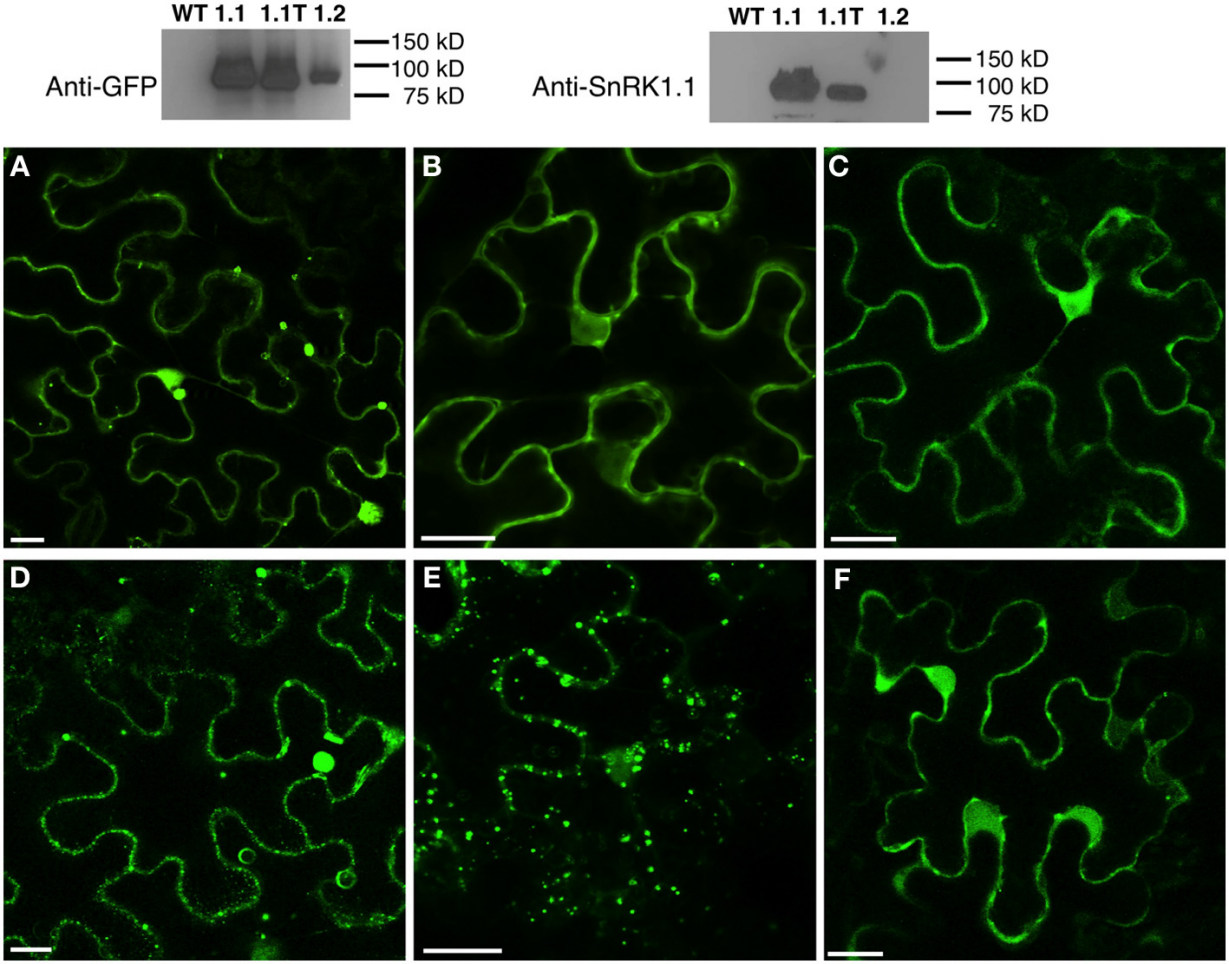
**Subcellular Location of SnRK1.1-, SnRK1.1T-, and SnRK1.2-GFP Proteins. Upper:** Leaves of *N. benthamiana* transiently expressing SnRK1.1-GFP, SnRK1.1T-GFP, and SnRK1.2-GFP were analyzed by protein blotting with the indicated antiserum. **Lower:** Single optical sections of *N. benthamiana* transiently expressing SnRK1.1-GFP **(A,D)**, SnRK1.1T-GFP **(B,E)**, and SnRK1.2-GFP **(C,F)**. A small section of mature leaves were removed and epidermal cells imaged using confocal microscopy at 0 min **(A–C)** and 30 min **(D–F)**. Scale Bar = 20 μm.

To investigate the subcellular location of SnRK1.1, SnRK1.1T, and SnRK1.2, we first expressed each construct in *N. benthamiana* leaves in transient expression assays. To ensure the patterns observed were not due to overexpression and off-target accumulation, we used confocal microscopy to image cells at 12, 24, 48, and 72 h after infiltration (data not shown). We found each SnRK1-GFP isoform was expressed and accumulated stably as measured by protein blotting (Figure 10, Top). 48 h after agro-infiltration, SnRK1.1-GFP localizes to the cytoplasm and nucleus, however the most striking signal is from large puncta that do not co-localize with chloroplasts (Figure 10A). To further investigate these puncta we transiently expressed SnRK1.1-GFP with mCherry organelle markers in *N. benthamiana*. The SnRK1.1-GFP puncta do not co-localize with Golgi, peroxisomes, or mitochondria (Supplemental Figures 5A–C). We found that SnRK1.1T-GFP localizes to the cytoplasm and nucleus as does SnRK1.2-GFP (Figures 10B,C, respectively). While performing localization experiments, it was observed that SnRK1.1T-GFP plants form very small puncta in addition to the prior nuclear and cytoplasmic signal. The small puncta first appear in cells near the cut margin of the leaf, and increased over time after cutting and preparing samples for microscopy (Figure 10E). Similar smaller puncta were observed in SnRK1.1-GFP cells, however they took on the order of 30 more minutes to appear (Figure 10D). No large accumulation of puncta were observed in SnRK1.2-GFP cells, although there were areas in these cells close to the plasma membrane with a spotty pattern that appear to be distinct from the smaller puncta seen in SnRK1.1T-GFP and SnRK1.1-GFP samples (Figure 10F). To determine the identity of these small puncta, co-localization experiments were performed with mCherry organelle markers. Small puncta from SnRK1-1T-GFP cells do not co-localize with Golgi, peroxisomal, or mitochondrial markers (Supplemental Figures 5D–F).

This pattern was confirmed in Arabidopsis with confocal imaging of homozygous progeny of two different overexpressing lines of SnRK1.1-GFP and SnRK1.1T-GFP plants. We found prominent large puncta in the SnRK1.1a line (Supplemental Figure 6A), and cytoplasmic localization in both lines (Supplemental Figures 6A,B). SnRK1.1a is the line with more abundant expression (Figure 6). In SnRK1.1T-GFP plants we found that GFP fluorescence was cytoplasmic. Interestingly, small puncta began to appear in SnRK1.1Ta cells 60 min after mechanical wounding (i.e., cutting of the leaf margin) (Supplemental Figure 5E). We did not see the consistent nuclear signal observed in *N. benthamiana* for either Arabidopsis SnRK1.1 construct (compare Figure 10 and Supplemental Figure 5). We could not detect reliable GFP fluorescence in any of our SnRK1.2:GFP lines, thus we conclude that the SnRK1.2-GFP protein is most likely below the level of detection using our confocal imaging conditions.

From both the *N. benthamiana* and Arabidopsis work we conclude that the SnRK1.1T isoform can localize to both the nucleus and cytoplasm, as well as being contained in small puncta of unknown origin. These puncta are stimulated by mechanical wounding and/or the stress of preparing samples, and may indicate that the SnRK1.1T isoform is dynamic and moves within the cell. The extra 23 amino acids on the N-terminus of SnRK1.1-GFP is associated with the unique appearance of larger puncta that also are not part of a known organelle. Lastly, the SnRK1.2 isoform has a similar nuclear and cytoplasmic location, but does not accumulate in small puncta after wounding.

## Discussion

The SnRK1 protein is a major regulator of plant growth and development, and when overexpressed it can re-program metabolism (Baena-Gonzalez et al., 2007; Jossier et al., 2009) and delay developmental transitions (Tsai and Gazzarrini, 2012a). Since most previous work has focused on the *SnRK1.1* gene, we sought to understand how *SnRK1.1* and *SnRK1.2* are regulated, and whether ectopic expression of each has the same or different consequences on plant growth and development. Our work provides key insights into spatial patterns of *SnRK1.1* and *SnRK1.2* expression, and also in the ability of *SnRK1.2* to impact plant growth when overexpressed.

We used promoter:GUS transgenic plants and quantitative PCR to show that *SnRK1.2* is much less abundantly expressed as compared to the *SnRK1.1* gene (Figures 1, 2). In addition, although *SnRK1.1* is broadly expressed in both shoot and root tissues, *SnRK1.2* expression is restricted to the hydathodes, cells at the base of leaf primordia, and some portions of vascular tissues. *SnRK1.2* is also expressed in roots throughout development past 5 d (Figures 1, 2). Given the previous biochemical and genetic studies by others showing that the SnRK1.2 is a protein kinase implicated in energy regulation (Baena-Gonzalez et al., 2007), we speculate that SnRK1.2 acts within a limited number of cells in the plant. We note here that this pattern of expression is similar to that from some genes involved in nutrient transport/sensing (Barker et al., 2000; Pilot et al., 2004). Our finding of restricted SnRK1.2 expression agrees with previous observations that SnRK1.1 is responsible for the major part of SnRK1 activity in Arabidopsis suspension cells (Jossier et al., 2009).

We also found that *SnRK1.2* expression is elevated by trehalose (Figures 2, 3). Careful work by others has shown that trehalose mediates its effects via elevation of trehalose 6 phosphate (T6P) levels within plant cells (Schluepmann et al., 2004), where T6P is thought to communicate sugar or stress status (Schluepmann et al., 2003; O'Hara et al., 2013; Lastdrager et al., 2014). Previous microarray experiments have shown that *SnRK1.2* expression is elevated by trehalose, which suggested that SnRK1.2 participates in the signaling pathway to sense sugar or stress (Schluepmann et al., 2004). Our results bring spatial information to the trehalose regulation of SnRK1.2, indicating that this induction occurs primarily in the root area closest to the root-shoot junction. We speculate that roots, a sink tissue, may have a special need for responding to trehalose and T6P, as trehalose stimulates accumulation of ~5-fold more starch in source tissues such as the cotyledons and a corresponding decrease in starch in root columella cells (Aghdasi et al., 2010). Interestingly T6P is a regulator of flowering time (Wahl et al., 2013) and can also act as an inhibitor of SnRK1.1 and SnRK1.2 enzyme activity (Zhang et al., 2009; Delatte et al., 2011). Although it seems counter intuitive for T6P to increase SnRK1.2 transcription and decrease SnRK1.2 activity, it should be noted that T6P inhibition of SnRK1 activity requires an intermediary factor (Zhang et al., 2009). Thus it is possible that T6P dependence on this intermediary factor allows T6P to elevate *SnRK1.2* transcription in the root, while decreasing activity elsewhere.

One of the most compelling reasons to study SnRK1 function in plants is the alteration of growth and development conferred by overexpression of SnRK1 (McKibbin et al., 2006; Baena-Gonzalez et al., 2007; Tsai and Gazzarrini, 2012a) (Supplemental Figure 4), which could be a useful tool for engineering desirable traits (Coello et al., 2011). Specifically, others have shown that overexpression of SnRK1.1 delays developmental transitions, such as the vegetative to reproductive transition, which manifests as a delay in time to flowering (Tsai and Gazzarrini, 2012a). This delay effectively decreases the size of SnRK1.1 overexpressors early in development, as measured by a smaller rosette width (Figure 9). Similarly, we found that SnRK1.1-GFP and SnRK1.1T-GFP plants flowered late and had smaller rosettes up to 28 d (Figures 8, 9). In contrast, overexpression of SnRK1.2, which was accompanied by increased levels of *SnRK1.2* RNA (Figure 6), resulted in early flowering (Figure 8), and larger rosettes and leaves prior to day 28 (Figure 9, Supplemental Figure 4). These data indicate that SnRK1.2, when overexpressed, has the opposite impact on flowering time as compared to SnRK1.1 overexpression. Previous biochemical studies have supported a similar enzyme activity for SnRK1.1 and SnRK1.2 proteins (Baena-Gonzalez et al., 2007; Jossier et al., 2009), and knockdown of SnRK1.1 and SnRK1.2 in Arabidopsis also support a redundant role for these two proteins. The mechanism for how overexpression of SnRK1.2 leads to a change in growth and shortens time to flowering is unknown at present. We speculate that the *SnRK1.2* gene, which is normally restricted in expression (in hydathodes, at the base of leaf primordia, and in vascular tissues within both seedling shoots and roots), could impact changes in flowering time gene regulation. As we see very little SnRK1.2:GFP protein accumulation in our overexpression lines, we suggest that a small difference in SnRK1.2 expression may have significant effects due to elevated protein kinase activity and impact on signaling. This concept of slight changes in kinase activity triggering significant downstream effects by amplifying signals has been discussed previously (Chock et al., 1980).

Another possibility for the seemingly opposite impact of SnRK1.1 and SnRK1.2 overexpression, is that SnRK1.1 and SnRK1.2 physically interact with different protein partners in the cell, and such interactors could be key for driving different biological outcomes in SnRK1 overexpression plants. In support of this, a recent query of String proteome-wide binary protein-protein interactions (Szklarczyk et al., 2011) indicated that SnRK1.1 and SnRK1.2 have some novel interactors. These include two different senescence-associated proteins, along with FUS3 and FUS5 for SnRK1.1, and JAZ3, the TOE2 transcription factor, and Starch Excess 4 (SEX4) for SnRK1.2. It is interesting to note that *sex4* mutants show a prolonged juvenile stage, and flower late (Matsoukas et al., 2013), thus this potentially novel SnRK1.2 interactor has a known connection to regulation of flowering time.

To further understand SnRK1.2 function, we performed subcellular localization studies using both a transient expression assay, and by examining transformed Arabidopsis. We found SnRK1.2 localized to both the nucleus and cytoplasm, which is the same location we and others have documented for SnRK1.1 (Lopez-Paz et al., 2009; Bitrian et al., 2011; Tsai and Gazzarrini, 2012a; Mohannath et al., 2014). An intriguing finding is that SnRK1.1T-GFP is stimulated by mechanical wounding to localize in small puncta (Figure 9, Supplemental Figure 6). These small puncta are not part of known organelles such as chloroplasts, mitochondria, peroxisomes, or the Golgi apparatus (Supplemental Figure 5). These puncta have been previously noted by others (Lopez-Paz et al., 2009; Bitrian et al., 2011; Tsai and Gazzarrini, 2012a), but our report is the first to connect these to a mechanical wounding stimulus. It is known that rice SnRK1.1T-GFP most likely moves between the nucleus and cytoplasm (Cho et al., 2012). We found SnRK1.1T in both the nucleus and cytoplasm in transient expression assays, but could not confirm the nuclear location in mature Arabidopsis leaves. Others reported SnRK1.1T-GFP in the chloroplast of stably transformed Arabidopsis (Fragoso et al., 2009). While we cannot rule out that some portion of SnRK1.1T-GFP localized to chloroplasts and it was below our limits of detection in our studies, our data strongly support the cytoplasm and small puncta as areas of the cell where SnRK1.1T-GFP accumulates to the highest degree. We speculate that the small puncta allow for movement of SnRK1.1T between compartments in response to stress. In addition, since we never observed SnRK1.2-GFP in small puncta or formation in response to wounding, we hypothesize that movement within the cell is unique to SnRK1.1 isoforms.

We also addressed the existence of a longer *SnRK1.1* cDNA that is predicted to encode a SnRK1.1 protein with 23 extra amino acids at the N-terminus. We found only minimal expression of this SnRK1.1 cDNA (Figure 5), and the SnRK1.1-GFP encoded by this cDNA had an additional subcellular location of accumulation within large puncta (Figure 9). SnRK1.1-GFP transiently expressed also localized to small puncta after wounding, although there was a delay in appearance of these puncta. Understanding what role, if any, this SnRK1.1 isoform plays in plants awaits purification of native SnRK1.1 isoforms and analysis via mass spectrometry.

In conclusion, the data reported here support a role for *SnRK1.2* as a spatially restricted *SnRK1* isoform that is capable of inducing early flowering when overexpressed. In addition, our results indicate an intriguing new possibility that certain stresses, such as mechanical wounding, induce movement or redistribution of SnRK1.1T protein in the cell.

### Conflict of interest statement

The authors declare that the research was conducted in the absence of any commercial or financial relationships that could be construed as a potential conflict of interest.

## Supplementary material

Supplemental Figure 1 **Amino acid alignment of SnRK1 proteins**. Predicted amino acid sequences of SnRK1.1, SnRK1.1T, and SnRK1.2 were aligned with ClustalW.

Supplemental Figure 2 **Anti-SnRK1 Antibody and Activity Assay. (A)** Purified SnRK1.1-V5 recombinant protein was separated by SDS-PAGE. **(B,D)** Immunoreactivity of anti-SnRK1.1 with native SnRK1.1. Plant extracts were made from wildtype Ler plants (WT), SnRK1.1.T-HA (1.1T-HA), and SnRK1.1 RNAi knock-down lines 10-1 and 10-2 (Baena-Gonzalez et al., 2007). Protein blots were probed with a 1:1500 dilution of purified anti-SnRK1.1 **(B)** or 1:2500 dilution of a commercial anti-HA antibody **(D)**. **(C)** Linearity of SnRK1 activity assay conditions. Means ± SE are presented.

Supplemental Figure 3 **Overexpression of SnRK1.1 in Ler**. Transgenic plants overexpressing SnRK1.1T-HA (SnRK1-Ox) and matched control WT (Ler) were grown under LD conditions.

Supplemental Figure 4 **Appearance and leaf size measurements of SnRK1 transgenic plants. (A)** 30 d wildtype and transgenic plants. **(B)** Leaf length and width measurements taken at 27 d.

Supplemental Figure 5 **Colocalization of SnRK-GFP with organelle markers**. Single optical sections of *N. benthamiana* transiently expressing SnRK1.1-GFP **(A–C)** and SnRK1.1T-GFP **(D,E)**. SnRK-GFP was co-expressed with a set of mCherry tagged organelle markers, golgi **(A,D)**, peroxisome **(B,E)**, and mitochondria **(C,F)**. Epidermal cells of mature leaves were imaged using confocal microscopy. Bar = 20 μm.

Supplemental Figure 6 **Subcellular localization of SnRK1.1 and SnRK1.1T in Arabidopsis**. Epidermal cells of mature leaves of soil grown Arabidopsis plants imaged with confocal microscopy. Two independent lines of SnRK1.1:GFP [1.1a **(A)** and 1.1b **(B)**] and SnRK1.1T:GFP [1.1Tb **(C)** and 1.1Ta **(D)**] were imaged immediately after cutting leaf sections. The two lines of SnRK1.2:GFP gave no detectable signal. Leaf sections were imaged again after 30 and 60 min. The SnRK 1.1Ta contain small fluorescent puncta ~60 min after wounding **(E)**. Bar = 20 μm.
