# Supplementary material for: Regulation of Sucrose non-Fermenting Related Kinase 1 genes in Arabidopsis thaliana
Source: Front Plant Sci. 2014 Jul 10;5:324. doi: 10.3389/fpls.2014.00324 (PMC4090914; doi:10.3389/fpls.2014.00324)
Supplement: Supplemental Figure 1 — Amino acid alignment of SnRK1 proteins. Predicted amino acid sequences of SnRK1.1, SnRK1.1T, and SnRK1.2 were aligned with ClustalW. [file Presentation1.PPTX]

## Slide 1
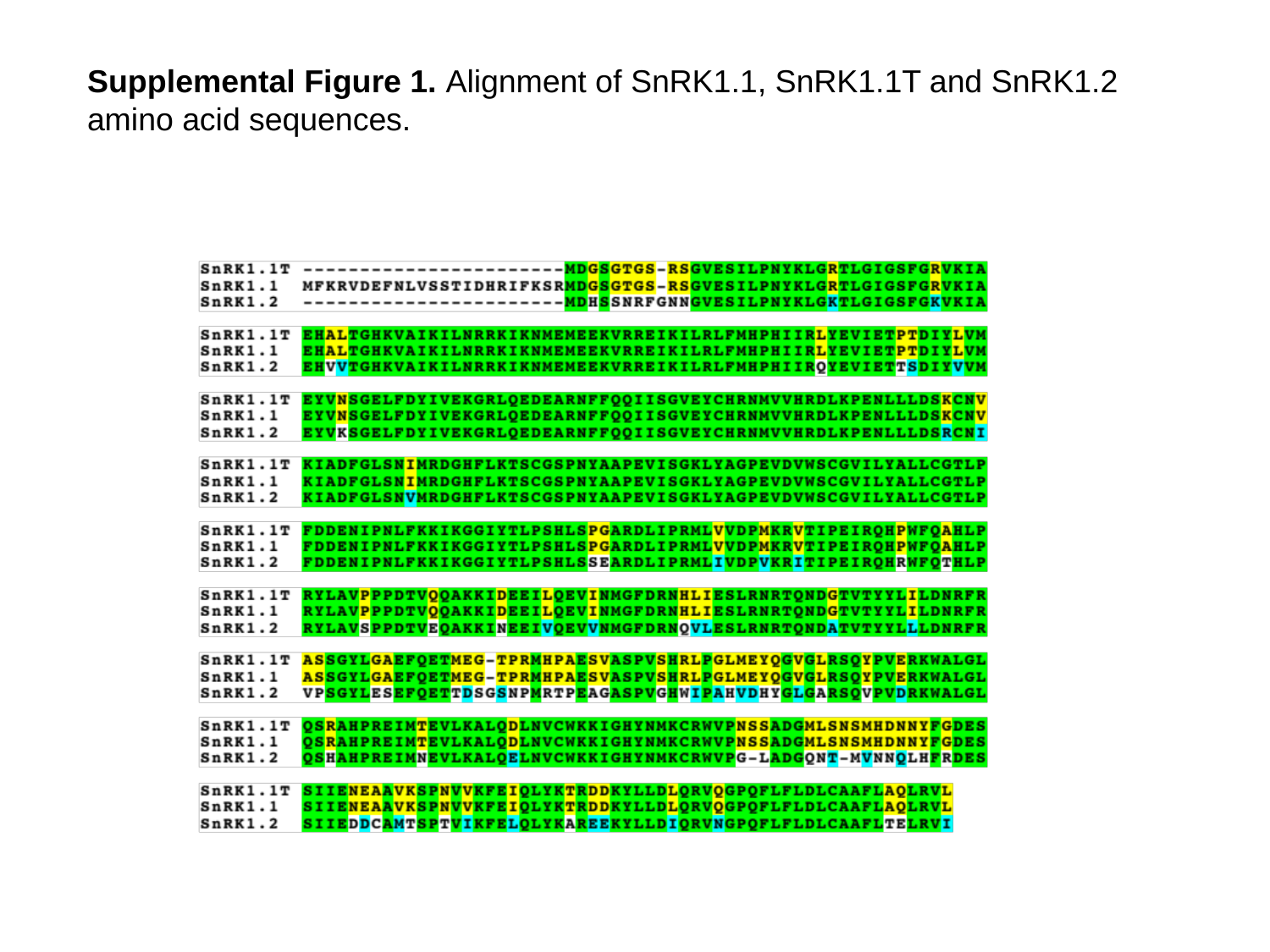

Supplemental Figure 1. Alignment of SnRK1.1, SnRK1.1T and SnRK1.2 amino acid sequences.
L

## Slide 2
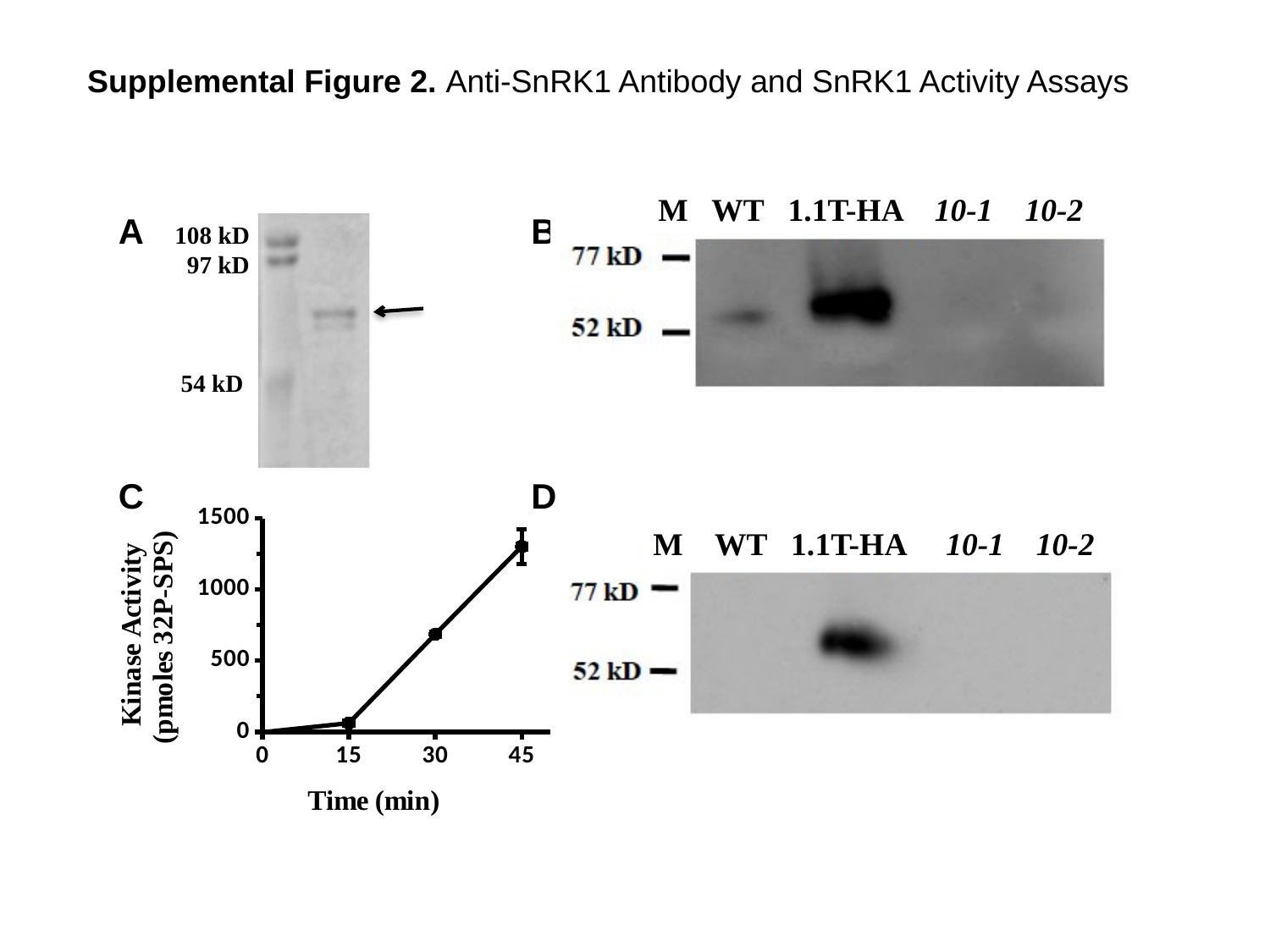

Supplemental Figure 2. Anti-SnRK1 Antibody and SnRK1 Activity Assays
M WT 1.1T-HA 10-1 10-2
A
B
108 kD
 97 kD
 54 kD
C
D
### Chart
| Category | Average |
|---|---|M WT 1.1T-HA 10-1 10-2

## Slide 3
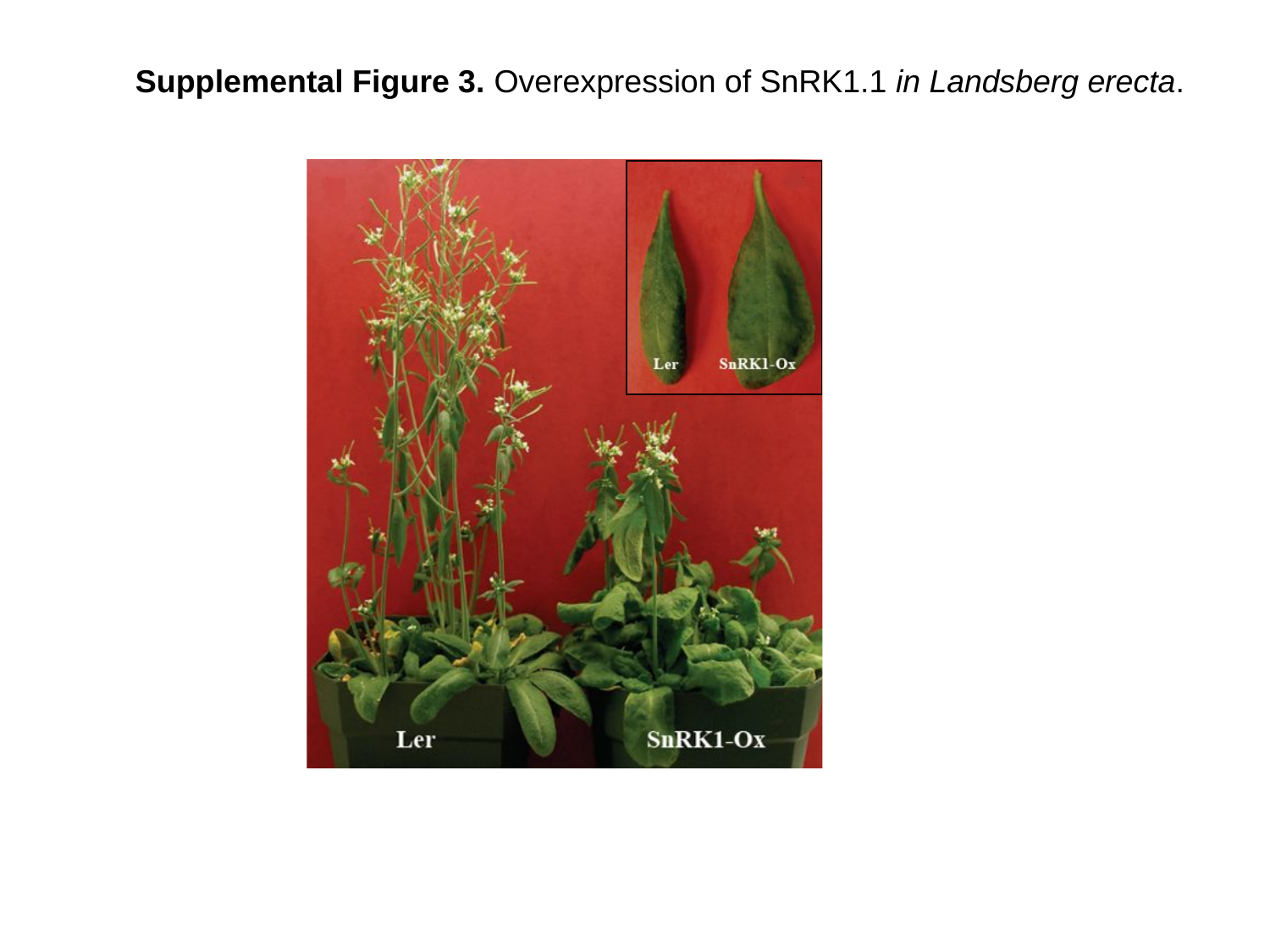

Supplemental Figure 3. Overexpression of SnRK1.1 in Landsberg erecta.

## Slide 4
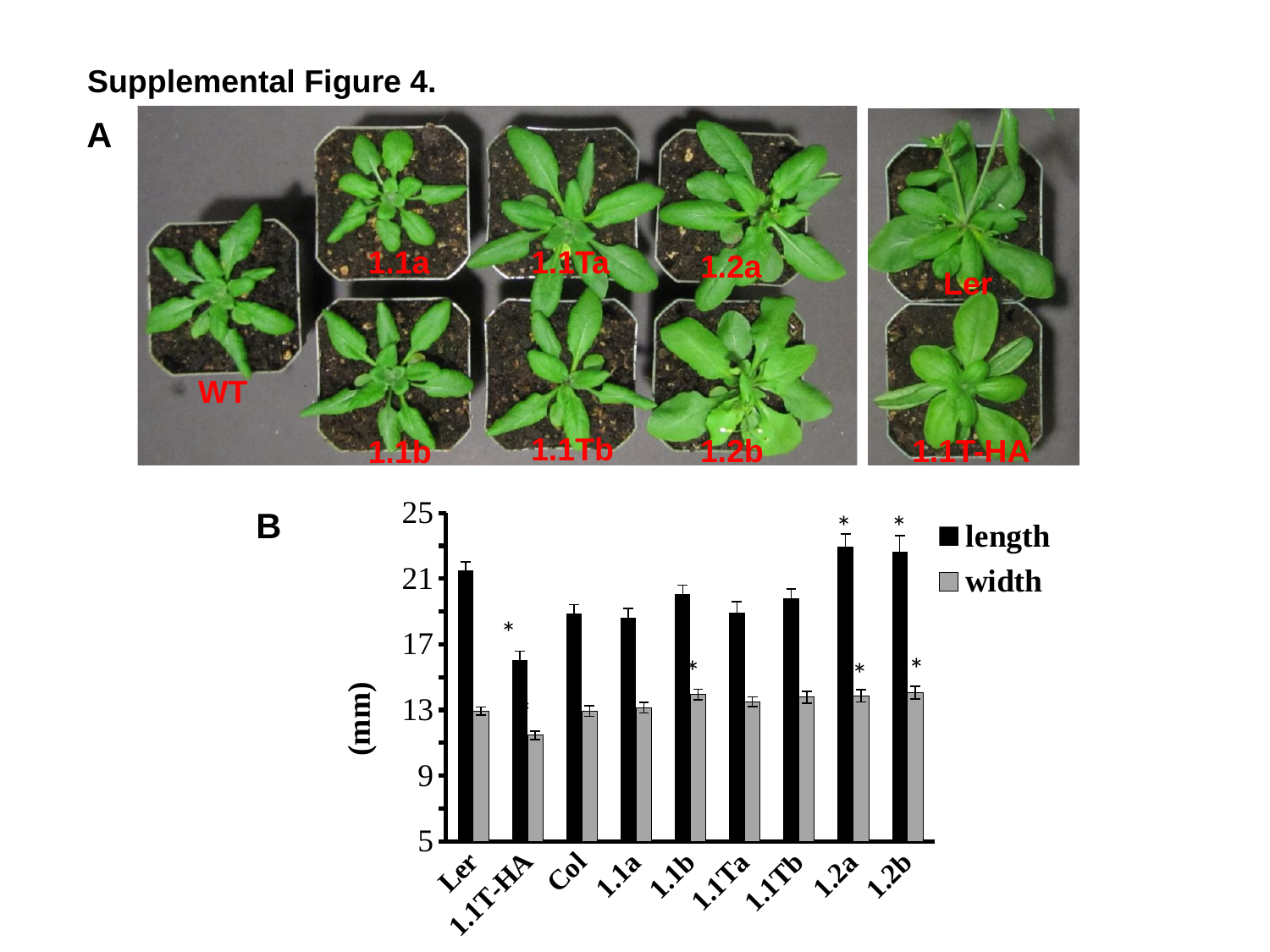

Supplemental Figure 4.
A
1.1a
1.1Ta
1.2a
Ler
WT
1.1Tb
1.2b
1.1T-HA
1.1b
### Chart
| Category | length | width |
|---|---|---|
| Ler | 21.5 | 12.94117647058824 |
| 1.1T-HA | 16.06451612903226 | 11.45161290322581 |
| Col | 18.86666666666667 | 12.93333333333333 |
| 1.1a | 18.61764705882353 | 13.14705882352941 |
| 1.1b | 20.05555555555556 | 13.94444444444445 |
| 1.1Ta | 18.94285714285714 | 13.51428571428571 |
| 1.1Tb | 19.80645161290322 | 13.7741935483871 |
| 1.2a | 22.97222222222218 | 13.86111111111111 |
| 1.2b | 22.63333333333332 | 14.06666666666667 |*
*
*
*
*
*
*
B

## Slide 5
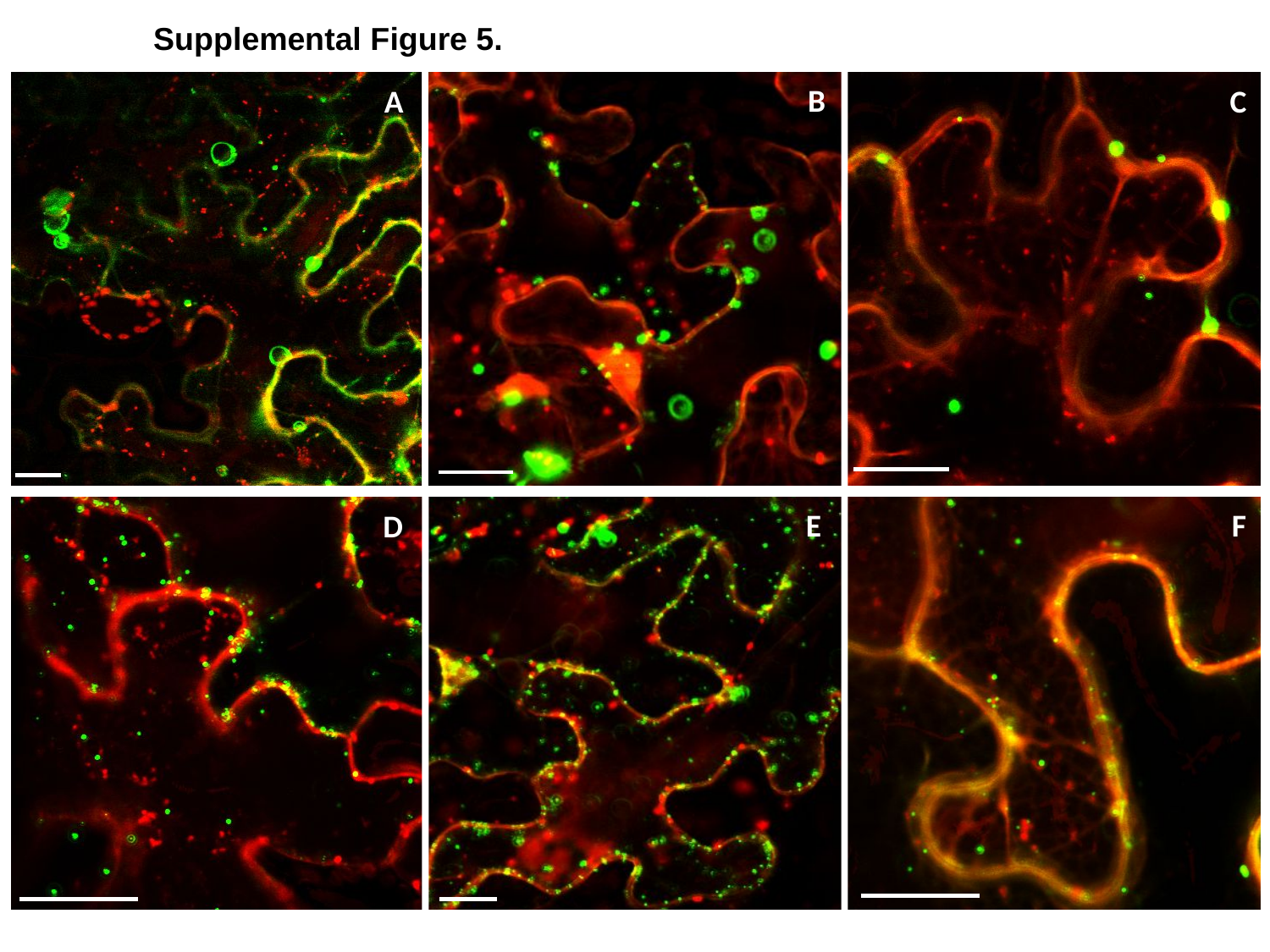

Supplemental Figure 5.
A
B
C
D
E
F

## Slide 6
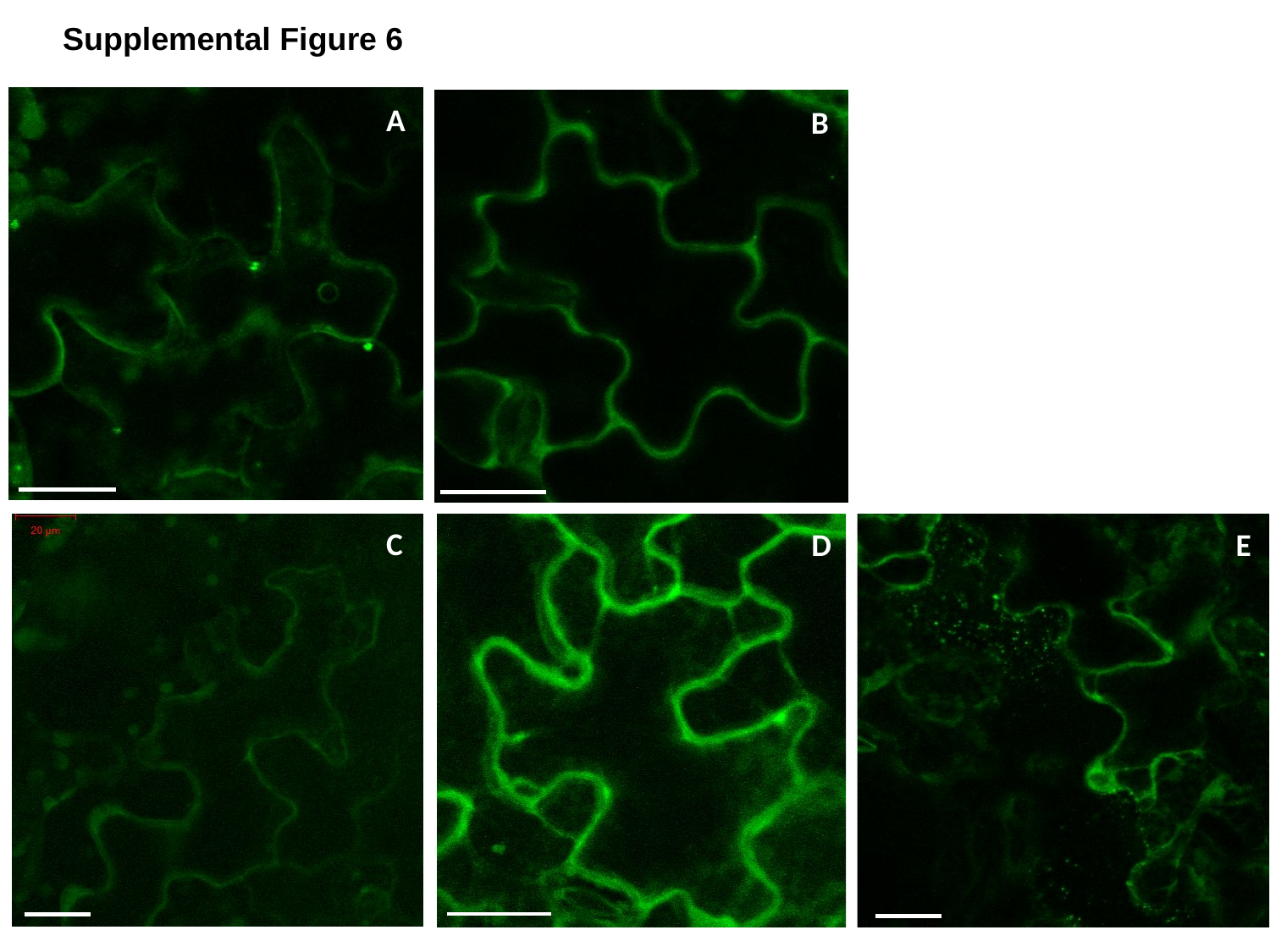

Supplemental Figure 6
1.1s3
B
A
B
E
C
D
E
