# Supplementary material for: Regulation of Sucrose non-Fermenting Related Kinase 1 genes in Arabidopsis thaliana
Source: Front Plant Sci. 2014 Jul 10;5:324. doi: 10.3389/fpls.2014.00324 (PMC4090914; doi:10.3389/fpls.2014.00324)
Supplement: Supplementary file 2 [file DataSheet1.DOCX]

**Supplemental Table 1.** Sequences and names of primers used in this work as given.

RT-PCR

qSnRK1.1*for*^1^ CCGAATTGGGGATAGTCTGAAAATTGC

qSnRK1.1*rev*^1^ CTCATCTACTCGTTTGAACATGAGAATTTAGCG

qSnRK1.1-All*for*^2^ GGAGATGGAGGAGAAAGTGAG

qSnRK1.1-All*rev*^2^ GAGTTCACATACTCCATGACAAG

qSnRK1.2*for* GAACTTCAGCTATACAAAGC

qSnRK1.2*rev* GCGCATAGATCCAAGAAG

qPEX4*for* CTTAACTGCGACTCAGGGAATCTTCTAAG

qPEX4*rev* TCATCCTTTCTTAGGCATAGCGGC

qGFP*for* GCACCATCTTCTTCAAGGACGA

qGFP*rev* TGTGGCTGTTGTAGTTGTACTCCAG

Cloning

SnRK1.1*for* ATGTTCAAACGAGTAGATGA

SnRK1.1*rev* GAGGACTCGGAGCTGAGCAAG

SnRK1.1T*for* ATGGATGGATCAGGCACAGG

SnRK1.1T*rev* GAGGACTCGGAGCTGAGCAAG

SnRK1.2*for* ATGGATCATTCATCAAATAG

SnRK1.2*rev* CACACGAAGCTCTGTAAG

GUS Cloning

SnRK1.1GUS*for* GATGACCTTTTTACTTGAGCTATTGAAG

SnRK1.1GUS*rev* GAGAATTTAGCGAGAATTAGGATCCCTTTTA

SnRK1.2GUS*for* GACAGATAAAAGCTTGGATTATAGAGATACAG

SnRK1.2GUS*rev* CGTCTTGGAGTAGATCCGAGAATC

^1^ Primers detect Accession #AY093170 and TAIR Accession At3g01090.2.

^2^ Primers detect Accession #AY093170 and TAIR Accessions At3g01090.1, At3g01090.2 and At3g01090.3.
